# Primary ciliogenesis is a crucial step for multiciliated cell determinism in the respiratory epithelium

**DOI:** 10.1111/jcmm.16729

**Published:** 2021-06-25

**Authors:** Randa Belgacemi, Zania Diabasana, Antony Hoarau, Xavier Dubernard, Jean‐Claude Mérol, Christophe Ruaux, Myriam Polette, Jeanne‐Marie Perotin, Gaëtan Deslée, Valérian Dormoy

**Affiliations:** ^1^ Université de Reims Champagne‐Ardenne, Inserm, UMR‐S1250, SFR CAP‐SANTE Reims France; ^2^ Department of otorhinolaryngology CHU Reims Hôpital Robert Debré Reims France; ^3^ Department of otorhinolaryngology Clinique Mutualiste La Sagesse Rennes France; ^4^ Department of biopathology CHU Reims Hôpital Maison Blanche Reims France; ^5^ Department of respiratory diseases CHU of Reims Hôpital Maison Blanche Reims France; ^6^ Present address: Lundquist Institute University of California Los Angeles Torrance CA USA

**Keywords:** airway epithelium, cell differentiation, cilia

## Abstract

The alteration of the mucociliary clearance is a major hallmark of respiratory diseases related to structural and functional cilia abnormalities such as chronic obstructive pulmonary diseases (COPD), asthma and cystic fibrosis. Primary cilia and motile cilia are the two principal organelles involved in the control of cell fate in the airways. We tested the effect of primary cilia removal in the establishment of a fully differentiated respiratory epithelium. Epithelial barrier integrity was not altered while multiciliated cells were decreased and mucous‐secreting cells were increased. Primary cilia homeostasis is therefore paramount for airway epithelial cell differentiation. Primary cilia‐associated pathophysiologic implications require further investigations in the context of respiratory diseases.

## 1 INTRODUCTION

Most primary cilia (PC) studies in respiratory research are restricted to development or repair processes. These solitary non‐mobile organelles act as sensors and molecular signalling hubs while motile cilia (MC) in differentiated epithelial cells are paramount for mucociliary clearance.^1^ We recently demonstrated the presence of PC in human airway epithelial undifferentiated cells during homeostasis and in chronic obstructive pulmonary disease (COPD).^2^ In addition, recent studies reported a decrease in multiciliated cells (MCC) in COPD patient‐derived bronchial and bronchiolar air‐liquid interface (ALI) cultures,^3^, ^4^ and we highlighted an abnormal cilia‐associated genomic signature in COPD patients (CiliOPD ^5^ ). As it was suggested that multiciliated cells (MCC) originated from primary ciliated cells (PCC),^6^ we investigated the consequences of PC removal on human airway epithelial cell (AEC) differentiation.

## 2 MATERIALS AND METHODS

Detailed materials and methods are provided in the Appendix S1 section.

## 3 RESULTS

**FIGURE 1:**
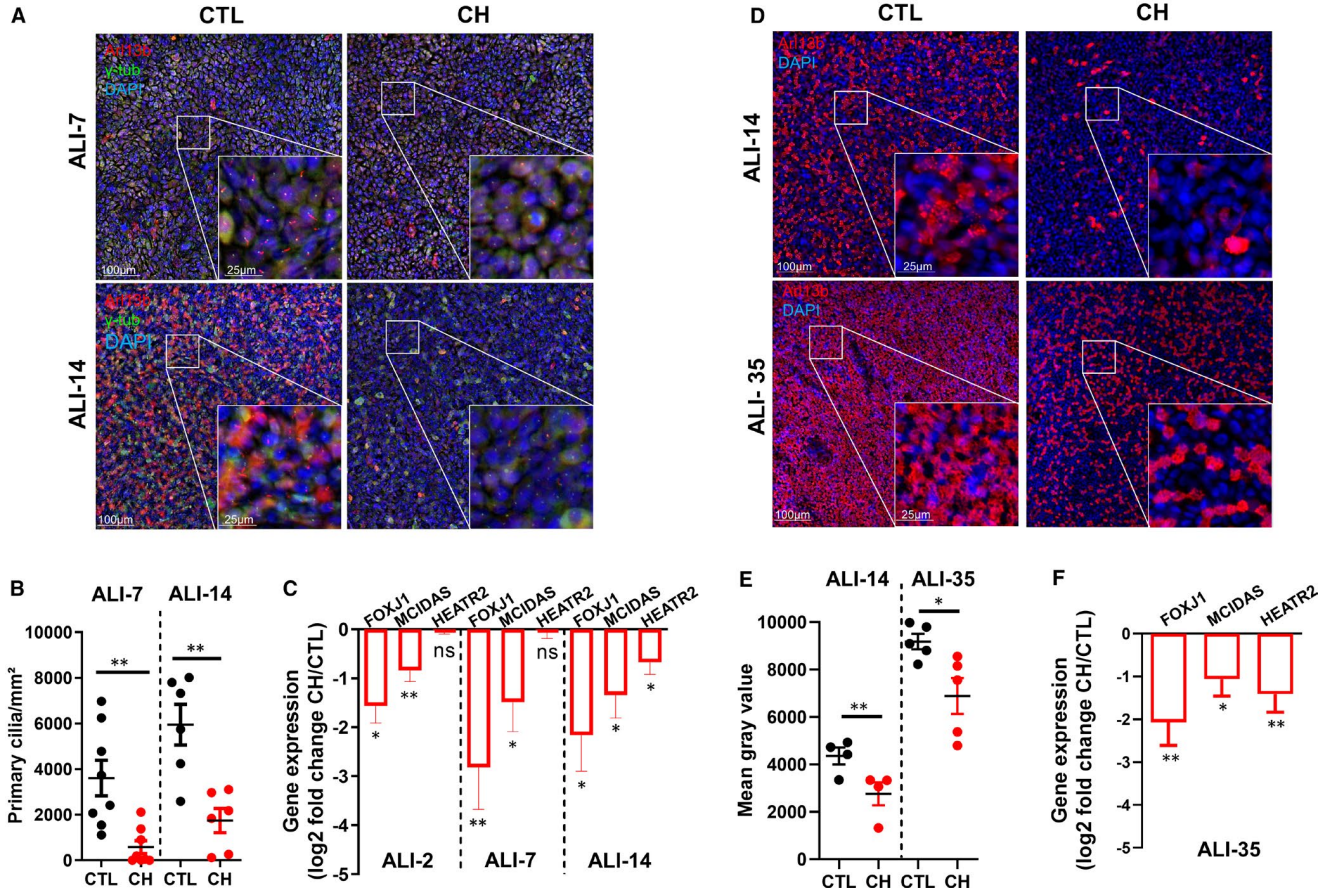
Loss of PC induces an alteration of motile ciliogenesis during AEC differentiation. A, Examples of micrographs taken from AEC cultures at ALI‐7 (upper panel) and ALI‐14 (bottom panel) showing PC (Arl13b, red; γ‐tubulin, green). Nuclei are stained in blue (DAPI). B, Dot plot (mean ±SEM) showing PC number per mm² at ALI‐7 (n = 8) and ALI‐14 (n = 6) in control (CTL) condition (black) and CH (red)‐treated cells. **P < 0.01 CH vs CTL. C, Histograms representing the assessment of fold‐change (log2) in the normalized expression to GAPDH during ALI cultures by RT‐qPCR (n = 11) for ciliogenesis markers FOXJ1, MCIDAS and HEATR2 at ALI‐2; ALI‐7 and ALI‐14. Results show mean ±SEM, **P < 0.005, *P < 0.05 CH vs CTL. D, Examples of micrographs taken from AEC cultures at ALI‐14 (left panel) and ALI‐35 (right panel) showing MC (Arl13b, red). Nuclei are stained in blue (DAPI). E, Dot plot (mean ±SEM) represents the mean grey values of MC‐associated fluorescence in CTL condition (black; n = 4) and CH‐treated cells (red; n = 5) at ALI‐14 and ALI‐35. **P < 0.01 CH vs CTL and *P < 0.05 CH vs CTL. F, Histograms representing the assessment of fold‐change CH/CTL (log2) in the normalized expression to GAPDH during ALI cultures by RT‐qPCR (n = 10) for ciliogenesis markers FOXJ1, MCIDAS and HEATR2 at ALI‐35. Results show mean ±SEM, **P < 0.005, *P < 0.05 CH vs CTL

First, we confirmed the deciliation induced by CH on ALI AEC. As it was shown that deciliation occurred in less than 24 hours post‐treatment,^7^ we maintained PC loss with daily treatment of CH. Considering that multiple PC per cell is a very rare event, we observed a sixfold decrease in PCC at ALI‐7 (3607 ± 778.5 PC/mm² in control (CTL) versus 582.3 ± 283.8 PC/mm² in CH‐treated cells) and a threefold decrease at ALI‐14 (5947 ± 891 PC/mm² in CTL versus 1745 ± 528.8 PC/mm² in CH‐treated cells) (Figure 1A and B). Key ciliogenesis‐associated genes such as FOXJ1, MCIDAS and HEATR2^8^ were significantly down‐regulated upon CH treatment as early as 2 days after the initiation of differentiation (Figure 1C).

We next investigated the impact of PC removal on MC establishment. As daily treatment with CH ultimately leads to cell death after 14 days of culture, we maintained PC inhibition with CH treatment every other day instead. In this condition, non‐differentiated AEC entered a 48‐hour cycle of ciliogenesis/deciliation for the duration of ALI cell culture.^7^ Chloral hydrate‐treated ALI cell cultures presented significantly less MCC than control ALI cell cultures with a 40% reduction at ALI‐14 and a 25% reduction at ALI‐35 (Figure 1D and E). The down‐regulation of key ciliogenesis‐associated genes persisted at ALI‐35 (Figure 1F). These findings suggest that PC are essential to establish a fully differentiated epithelium with MCC.

**FIGURE 2:**
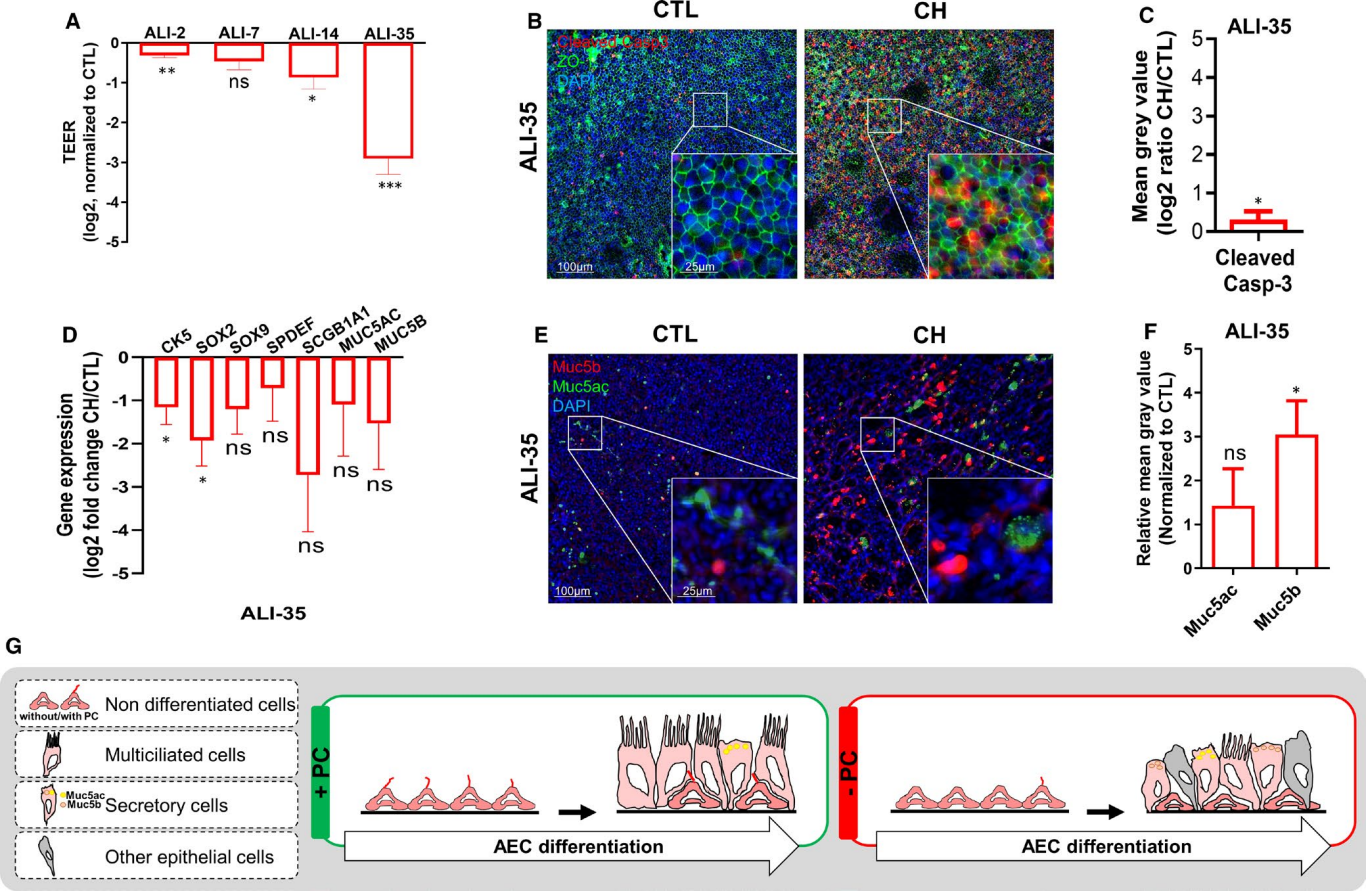
Loss of PC induces global epithelial remodelling during AEC differentiation. A, Histograms representing the TEER (log2, normalized to CTL, n = 6) of CH‐treated ALI cultures (n = 6). Results show mean ±SEM, ***P < 0.001, **P < 0.01, *P < 0.05 CH vs CTL. B, Examples of micrographs taken from AEC cultures at ALI‐35 showing cleaved caspase‐3 (red) and Zonula occludens‐1 (ZO1, green). Nuclei are stained in blue (DAPI). C, Histogram representing mean grey values of the cleaved caspase‐3‐associated fluorescence of CH‐treated AEC (n = 3) in log 2 ratio CTL/CH at ALI‐35. *P < 0.05 CH vs CTL. D, Histograms representing the assessment of fold‐change (log2) in the normalized expression to GAPDH during ALI cultures by RT‐qPCR (n = 11) for non‐differentiated cell markers (CK5, SOX2, SOX9), and secretory cell markers (SPDEF, SCGB1A1, MUC5AC, MUC5B) at ALI‐35. Results show mean ±SEM, *P < 0.05 CH vs CTL. E, Examples of micrographs taken from AEC cultures at ALI‐35 showing mucins (Muc5b, red; Muc5ac, green). Nuclei are stained in blue (DAPI). F, Histogram representing the relative mean grey values of the mucins‐associated fluorescence normalized to CTL at ALI‐35 of CH‐treated cells for Muc5b and Muc5ac (n = 9). *P < 0.05 CH vs CTL. G, Illustration summarizing in vitro AEC remodelling upon PC inhibition

To further address the role of PC in AEC differentiation, we next analysed epithelial barrier integrity. Transepithelial electrical resistance (TEER) was significantly reduced during differentiation with a sixfold decrease in ALI‐35 in CH‐treated cells (Figure 2A). Therefore, we evaluated tight junction assembly and apoptosis. We did not find any differences in the junctional network whereas apoptosis was slightly increased (Figure 2B and C). This suggests that the loss of TEER is likely caused by a reduction in epithelial height and a lack of differentiation rather than a permeability issue. To further investigate the global epithelial differentiation programming, we assessed the transcript levels of markers for non‐differentiated cells (CK5, SOX2, SOX9) and secretory cells (SPDEF, SCGB1A1, MUC5AC, MUC5B). We observed a significant decrease in non‐differentiated cell markers, and a trend towards the decreased expression of all the others (Figure 2D). Considering that an increase in mucous‐secreting cells is a common feature of several lung diseases, we also evaluated the production of the two main mucins in the lung: Muc5ac and Muc5b. Interestingly, Muc5b‐secreting cells but not Muc5ac‐secreting cells showed a threefold increase in CH‐treated ALI cultures (Figure 2E and F). These findings suggest that PC are essential to warrant epithelial cell integrity and orientate cell fate determination during AEC differentiation (Figure 2G).

## 4 DISCUSSION

To our knowledge, we provided the first experimental demonstration that PC directly orchestrate MCC differentiation and are crucial for AEC homeostasis. We confirmed previous in vitro observations of transient PC ^6^ but in contrast, we detected non‐transient PC in human adult non‐differentiated AEC during differentiation. This was consistent with our previous report on human lung adult tissues.^2^

Deciliation has been indicted in epithelial remodelling,^7^ and the cell populations obtained after 35 days of culture upon primary ciliogenesis inhibition described two major features of respiratory diseases characterized by remodelled epithelium including a decrease in MCC and an increase in goblet cells.^9^ Our findings highlight the role of PC in the process of AEC differentiation. This is particularly important as a recent study demonstrated the presence of a hybrid cilium in MCC with PC features,^10^ and PC functions are discussed in several respiratory diseases including COPD, asthma and idiopathic pulmonary fibrosis.^2^, ^11^, ^12^

Given that PC are involved in many signalling pathways, a better understanding of their involvement in lung physiology and pathologies may help identify crucial molecular actors to expand diagnosis, prognosis and therapeutics. Hedgehog pathway appears as the first candidate that has been connected to PC during lung development,^13^ and has been associated with AEC differentiation and lung diseases.^14^, ^15^ Two other crucial developmental signalling pathways, the canonical Wnt and Notch, have also been highlighted in human AEC remodelling and were further associated with COPD features.^16^, ^17^ Therefore, integrating the key players of organogenesis in the context of the global cilia‐associated alterations in lung diseases may pave the way towards the identification of promising biomarkers and targeted therapies.

There are two main limitations to our study. First, experimental approaches to reach deciliation are limited to gene silencing^18^ or pharmacological treatments with their associated caveats.^19^ Although CH targets highly stabilized microtubules (ie PC), non‐stabilized microtubules may also be impacted. Second, we used AEC isolated from nasal polyps as a surrogate of respiratory epithelia. It will be important to confirm our results on bronchial and bronchiolar epithelial cells.

In conclusion, we have shown that primary ciliogenesis is a crucial step for MCC determinism in AEC differentiation. We propose to include PC as important organelles to investigate in the context of respiratory research. In addition, the exploration of signalling pathways related to PC in the adult lung may provide novel perspectives to understand lung diseases associated with cilia alterations.

## CONFLICT OF INTEREST

Dr Ruaux reports grants and personal fees from Sanofi‐Aventis outside the submitted work. Dr Deslée reports personal fees from Nuvaira, personal fees from BTG/PneumRx, personal fees from Chiesi, personal fees from Boehringer and personal fees from Astra Zeneca, outside the submitted work. Dr Dormoy reports personal fees from Chiesi outside the submitted work.

## AUTHOR CONTRIBUTION

**Randa Belgacemi:** Conceptualization (equal); Formal analysis (equal); Investigation (equal); Writing‐original draft (equal); Writing‐review & editing (equal). **Zania Diabasana:** Formal analysis (supporting); Investigation (supporting); Writing‐review & editing (supporting). **Antony Hoarau:** Formal analysis (equal); Investigation (equal); Writing‐review & editing (supporting). **Xavier Dubernard:** Formal analysis (equal); Resources (equal); Writing‐review & editing (supporting). **Jean‐Claude Mérol:** Formal analysis (equal); Resources (equal); Writing‐review & editing (supporting). **Christophe Ruaux:** Formal analysis (equal); Resources (equal); Writing‐review & editing (supporting). **Myriam Polette:** Formal analysis (equal); Investigation (equal); Writing‐review & editing (equal). **Jeanne‐Marie Perotin:** Formal analysis (equal); Investigation (equal); Methodology (equal); Resources (equal); Writing‐original draft (equal); Writing‐review & editing (equal). **Gaëtan Deslée:** Formal analysis (equal); Investigation (equal); Resources (lead); Supervision (equal); Writing‐original draft (equal); Writing‐review & editing (equal). **Valérian Dormoy:** Conceptualization (lead); Formal analysis (lead); Funding acquisition (lead); Investigation (lead); Methodology (lead); Project administration (lead); Supervision (lead); Writing‐original draft (lead); Writing‐review & editing (lead).

## 5 ETHICS APPROVAL AND CONSENT TO PARTICIPATE

The study was approved by the institutional review board of the University Hospital of Reims, France (IRB Reims‐CHU 20 110 612), and was conducted in accordance with the ethical guidelines of the Declaration of Helsinki. All patients gave their written informed consent prior to inclusion in the study.

## Supporting information

Appendix S1

## Data Availability

The data that support the findings of this study are available from the corresponding author upon reasonable request.
