# Supplementary material for: Primary ciliogenesis is a crucial step for multiciliated cell determinism in the respiratory epithelium
Source: J Cell Mol Med. 2021 Jun 25;25(15):7575–9. doi: 10.1111/jcmm.16729 (PMC8335676; doi:10.1111/jcmm.16729)
Supplement: Supplementary file 1 — Appendix S1 [file JCMM-25-7575-s001.docx]

**Primary ciliogenesis is a crucial step for multiciliated cells determinism in the respiratory epithelium**

Randa Belgacemi, Zania Diabasana, Antony Hoarau, Xavier Dubernard, Jean-Claude Mérol, Christophe Ruaux, Myriam Polette, Jeanne-Marie Perotin, Gaëtan Deslée, Valérian Dormoy

**Supporting Information**

[**1.** **MATERIALS AND METHODS** 1](#_Toc72404415)

[**1.1.** **Human primary AEC cultures** 1](#_Toc72404416)

[**1.2.** **RT-qPCR analyses** 1](#_Toc72404417)

[**1.3.** **Whole-mount immunofluorescent stainings** 2](#_Toc72404418)

[**1.4.** **TEER measurements** 2](#_Toc72404419)

[**1.5.** **Statistics** 2](#_Toc72404420)

[**2.** **Supplementary Table** 3](#_Toc72404421)

[**Table S1. List of primers** 3](#_Toc72404422)

[**3.** **ADDITIONAL REFERENCES** 4](#_Toc72404423)

1. **MATERIALS AND METHODS**
   1. **Human primary AEC cultures**

Human primary AEC were obtained from nasal polyps (18 donors) to establish air-liquid interface (ALI) cultures as described by us and others [1–7]. The cells were dissociated by overnight pronase incubation (0.5mg/mL, Sigma-Aldrich). 200,000 cells were seeded on 12-well plates containing 0.4µm Transwells (Corning, Fisher Scientifc) coated with 0.3 mg/mL collagen type-IV (Sigma-Aldrich). PneumaCult-EX media (StemCell) was used for initial proliferation in apical and basal chambers. Upon reaching cell confluency, the apical medium was removed and PneumaCult-ALI (StemCell) medium was used in basal chamber. Culture medium was changed three times a week and cells kept in incubators at 37°C, 5% CO2. We used chloral hydrate (CH) treatment in ALI cultures to interfere with stable microtubules organization resulting in PC loss [8] [9]. Chloral hydrate (C8383, Sigma-Aldrich, 4 mM) was added to culture medium from the initiation of cell differentiation (ALI switch) either every day up to 14 days or every other day up to 35 days and cells were collected every 7 days for 35 days.

- 1. **RT-qPCR analyses**

Total RNA from AEC cultures was isolated by High Pure RNA isolation kit (Roche Diagnostics) and 250ng was reverse-transcribed into cDNA by Transcriptor First Stand cDNA Synthesis kit (Roche Diagnostics). Quantitative PCR reactions were performed with fast Start Universal Probe Master kit and UPL-probe system in a LightCycler-480 Instrument (Roche Diagnostics) as recommended by the manufacturer. Transcript expression levels were analyzed by RT-qPCR for PC and MC associated markers (FOXJ1, MCIDAS, HEATR2; [9]), basal cell markers (CK5, SOX2, SOX9), and secretory cell markers (SPDEF, SCGB1A1, MUC5AC, MUC5B). Specific primers (Eurogentec) are detailed in **Table S1**. Results for all expression data regarding transcripts were normalized to the expression of the house-keeping gene GAPDH. Relative gene expression was assessed by the ΔΔCt method [10] and expressed as fold change (log2, CH vs control) when indicated.

- 1. **Whole-mount immunofluorescent stainings**

Ciliated-(Arl13b positive; 17711-1-ap, ProteinTech), mucous-secreting-(Muc5ac- and Muc5b-positive; NBP-215196, Novus Biologicals, and E-AB-15988, Elabscience), and apoptotic-(cleaved caspase 3 positive; NB100-56708, Novus Biologicals)cells were quantified after immunofluorescent staining as previously described [1]. Concerning cilia, we assessed the number of ciliated cells (harbouring either one PC or the bundle of motile cilia). In addition, γ-tubulin (T6567, Merck) and ZO-1 (61-7300, ThermoFisher Scientific) antibodies were used. Micrographs were acquired by AxioImageur Zeiss (20xPh) with ZEN software (8.1, 2012) and processed with ImageJ.

- 1. **TEER measurements**

Transepithelial electrical resistance (TEER) was evaluated at ALI-2, ALI-7, ALI-14, and ALI-35 using an EVOM2 resistance meter with an STX2 electrode (World Precision Instruments Hitchin) at room temperature. The electrode was equilibrated in PnC-ALI for 1 h at room temperature before measurement. One mL PnC-ALI was added to the apical compartment and triplicate measurements were performed per sample. Data were corrected for blank values and area. Average resistance was subtracted from the measured value of every well according to data acquired on cell-free permeable supports and results are presented as log2 (normalized to non-treated cells) of the resistance per surface (Ω x cm²).

- 1. **Statistics**

Quantitative data in two groups were compared with Student’s test. Results with two-sided p-value ≤0.05 were considered significant. Data were represented with GraphPad software Inc. Prism Version 8, US.

1. **Supplementary Table**

**Table S1. List of primers**

| **GENES** | **Forward sequence** | **Reverse sequence** |
| --- | --- | --- |
| **FOXJ1** | 5’-CAGATCCCACCTGGCAGA-3’ | 5’-CGTACTGGGGGTCAATGC-3’ |
| **MCIDAS** | 5’-CATCTGCCCCAACAGAATG-3’ | 5’-GATCCTCGTACACCGACACC-3’ |
| **HEATR2** | 5’-ATCCTGTCCACCGTGCTG-3’ | 5’-CCAGGATGTCCTTTGTCACC-3’ |
| **CK5** | 5’-TTCATGAAGATGTTCTTTGATGC-3’ | 5’-AGGTTGCGGTTGTTGTCC-3’ |
| **SOX2** | 5’-ATGGGTTCGGTGGTCAAGT-3’ | 5’-GGAGGAAGAGGTAACCACAGG-3’ |
| **SOX9** | 5’-CGGAGGAAGTCGGTGAAG-3’ | 5’-GGGAGATGTGCGTCTGCT-3’ |
| **SPDEF** | 5’-GCACCAGGCAGCTAACAGA-3’ | 5’-GGGATACGCTGCTCAGACC-3’ |
| **SCGB1A1** | 5’-CTCACCCTGGTCACACTGG-3’ | 5’-CTGAAAGCTCGGGCAGAT-3’ |
| **MUC5AC** | 5’-CACGTCCCCTTCAATATCCA-3’ | 5’-GGCCCAGGTCTCACCTTT-3’ |
| **MUC5B** | 5’-GTACAATGGCACCTTCTACGG-3’ | 5’-CTGACATTGCACCGTTGG-3’ |
| **GAPDH** | 5′-ACCAGGTGGTCTCCTCTGAC-3′ | 5′-TGCTGTAGCCAAATTCGTTG-3′ |

1. **ADDITIONAL REFERENCES**

1. Belgacemi R, Luczka E, Ancel J, Diabasana Z, Perotin J-M, Germain A, Lalun N, Birembaut P, Dubernard X, Mérol J-C, Delepine G, Polette M, Deslée G, Dormoy V. Airway epithelial cell differentiation relies on deficient Hedgehog signalling in COPD. *EBioMedicine* 2020; 51: 102572.

2. Adam D, Roux-Delrieu J, Luczka E, Bonnomet A, Lesage J, Mérol J-C, Polette M, Abély M, Coraux C. Cystic fibrosis airway epithelium remodelling: involvement of inflammation. *J. Pathol.* 2015; 235: 408–419.

3. Jiang D, Schaefer N, Chu HW. Air-Liquid Interface Culture of Human and Mouse Airway Epithelial Cells. *Methods Mol. Biol. Clifton NJ* 2018; 1809: 91–109.

4. Schamberger AC, Staab-Weijnitz CA, Mise-Racek N, Eickelberg O. Cigarette smoke alters primary human bronchial epithelial cell differentiation at the air-liquid interface. *Sci. Rep.* 2015; 5: 8163.

5. Müller L, Brighton LE, Carson JL, Fischer WA, Jaspers I. Culturing of human nasal epithelial cells at the air liquid interface. *J. Vis. Exp. JoVE* 2013; .

6. Pezzulo AA, Starner TD, Scheetz TE, Traver GL, Tilley AE, Harvey B-G, Crystal RG, McCray PB, Zabner J. The air-liquid interface and use of primary cell cultures are important to recapitulate the transcriptional profile of in vivo airway epithelia. *Am. J. Physiol. Lung Cell. Mol. Physiol.* 2011; 300: L25-31.

7. Ruiz García S, Deprez M, Lebrigand K, Cavard A, Paquet A, Arguel M-J, Magnone V, Truchi M, Caballero I, Leroy S, Marquette C-H, Marcet B, Barbry P, Zaragosi L-E. Novel dynamics of human mucociliary differentiation revealed by single-cell RNA sequencing of nasal epithelial cultures. *Dev. Camb. Engl.* 2019; .

8. Khan NA, Willemarck N, Talebi A, Marchand A, Binda MM, Dehairs J, Rueda-Rincon N, Daniels VW, Bagadi M, Thimiri Govinda Raj DB, Vanderhoydonc F, Munck S, Chaltin P, Swinnen JV. Identification of drugs that restore primary cilium expression in cancer cells. *Oncotarget* 2016; 7: 9975–9992.

9. Praetorius HA, Spring KR. Removal of the MDCK cell primary cilium abolishes flow sensing. *J. Membr. Biol.* 2003; 191: 69–76.

10. Ancel J, Belgacemi R, Perotin J-M, Diabasana Z, Dury S, Dewolf M, Bonnomet A, Lalun N, Birembaut P, Polette M, Deslée G, Dormoy V. Sonic hedgehog signalling as a potential endobronchial biomarker in COPD. *Respir. Res.* 2020; 21: 207.
